# Supplementary material for: Fairness in Cardiac Magnetic Resonance Imaging: Assessing Sex and Racial Bias in Deep Learning-Based Segmentation
Source: Front Cardiovasc Med. 2022 Apr 7;9:859310. doi: 10.3389/fcvm.2022.859310 (PMC9021445; doi:10.3389/fcvm.2022.859310)
Supplement: Supplementary file 1 [file Data_Sheet_1.pdf]

Supplementary Material

1 Supplementary List 1. Exclusion criteria used for the selection of healthy volunteers from the UK Biobank database.

Medical conditions: Adrenocortical insufficiency/Addison's disease, Alcoholic liver disease/alcoholic cirrhosis, Anaemia, Angina, Ankylosing spondylitis, Anorexia/bulimia/other eating disorder, Antiphospholipid syndrome, Aortic aneurysm, Aortic regurgitation/incompetence, Aortic stenosis, Aplastic anaemia, Asthma, Atrial fibrillation, Atrial flutter, Bronchiectasis, Cardiomyopathy, Chronic obstructive airways disease, Clotting disorder/excessive bleeding, Connective tissue disorder, Crohn's disease, Diabetes insipidus, Diabetic eye disease, Diabetic neuropathy/ulcers, Doctor diagnosed bronchiectasis, Emphysema, Emphysema/chronic bronchitis, Fibrosing alveolitis/unspecified alveolitis, Gestational diabetes, Gestational diabetes, Gestational hypertension/pre-eclampsia, Giant cell/temporal arteritis, Glomerulonephritis, Grave's disease, Haemochromatosis, Haemophilia, Heart arrhythmia, Heart attack/myocardial infarction, Heart failure/pulmonary oedema, Heart valve problem/heart murmur, Heart/cardiac problem, Hereditary/genetic haematological disorder, Hyperaldosteronism/Conn's syndrome, Hyperprolactinaemia, Hyperthyroidism/thyrotoxicosis, Hypertrophic cardiomyopathy, Hypopituitarism, Hypothyroidism / myxoedema, IgA nephropathy, Inflammatory bowel disease, Interstitial lung disease, Iron deficiency anaemia, Irregular heartbeat, Kidney nephropathy, Leg claudication/intermittent claudication, Liver failure/cirrhosis, Low platelets/platelet disorder, Lymphoedema, Microscopic polyarteritis, Miscarriage, Mitral regurgitation/incompetence, Mitral valve disease, Mitral valve prolapse, Monoclonal gammopathy/not myeloma, Myeloproliferative disorder, Myocarditis, Myositis/myopathy, Nephritis, Neutropenia/lymphopenia, Other respiratory problems, Pericardial effusion, Pericardial problem, Pericarditis, Peripheral vascular disease, Pernicious anaemia, Pleural effusion, Pleural plaques (not known asbestosis), Polycythaemia vera, Polymyalgia rheumatica, Polymyositis, Pulmonary embolism +/- DVT, Renal failure not requiring dialysis, Renal/kidney failure, Respiratory failure, Retinal artery/vein occlusion, Rheumatic fever, Sarcoidosis, Sick sinus syndrome, Sickle cell disease, Sjogren's syndrome/sicca syndrome, Sleep apnoea, Stroke, Supraventricular tachycardia, Surgery/amputation of leg above the knee, Surgery/amputation of leg below the knee, Surgery/amputation of toe, Systemic lupus erythematosus, Transient ischaemic attack, Ulcerative colitis, Vasculitis, Wagner's granulomatosis, Wolff-Parkinson-White syndrome.

Medication: Hormone replacement therapy.

Symptoms: Shortness of breath walking on level ground, Chest pain due to walking ceases when standing still, Chest pain when walking uphill or hurrying, Unable to walk up hills or to hurry.

Supplementary Figure 1. CONSORT flow chart.

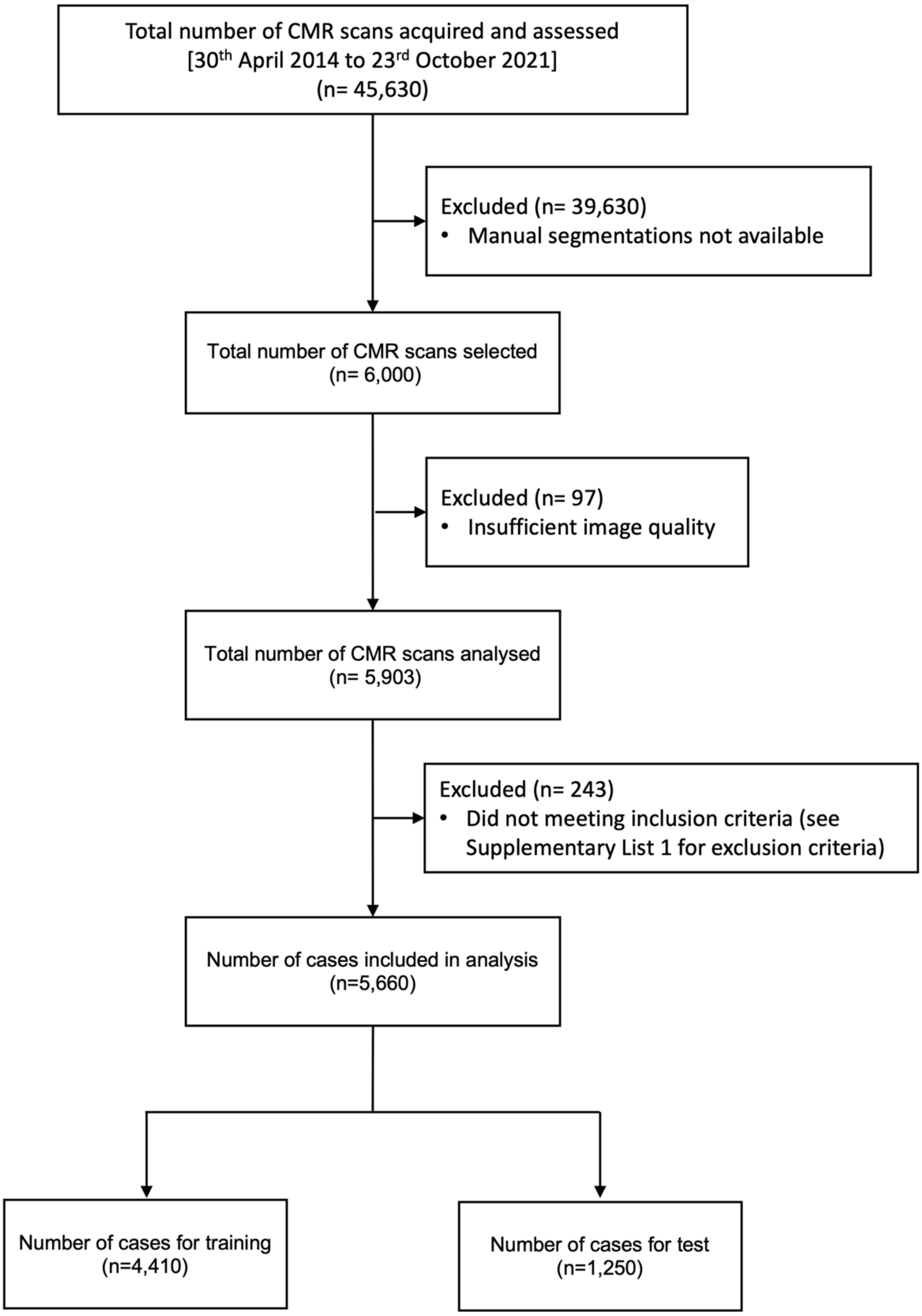

Supplementary Figure 2. Examples of frames from cine CMR sequence and their associated ground truth segmentations, as well as examples of predicted segmentations for each racial group.

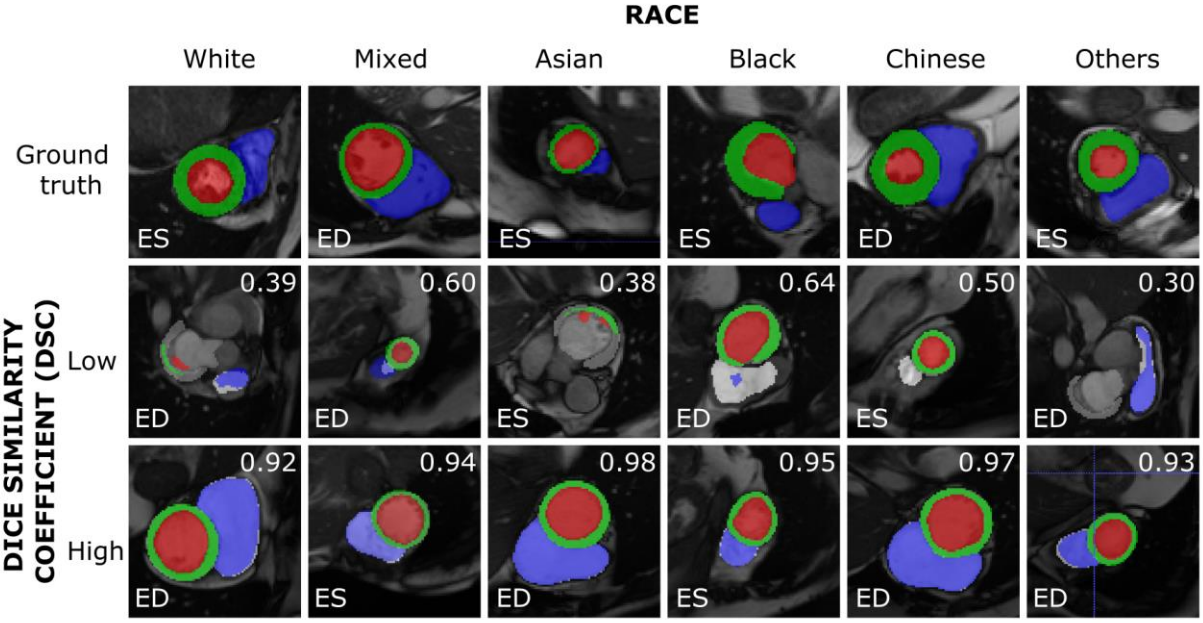

Top row: Illustration of sample frames from cine CMR sequences and their associated ground truth segmentations at ED and ES for the different racial groups. Middle and bottom row: Other sample frames showing predicted segmentation results at ED for each racial group with high (middle row) and low (bottom row) Dice similarity coefficient (DSC). This figure was partially adapted from (14).

Supplementary Figure 3. Bland-Altman plots of clinical measures between automated measurement and manual measurement.

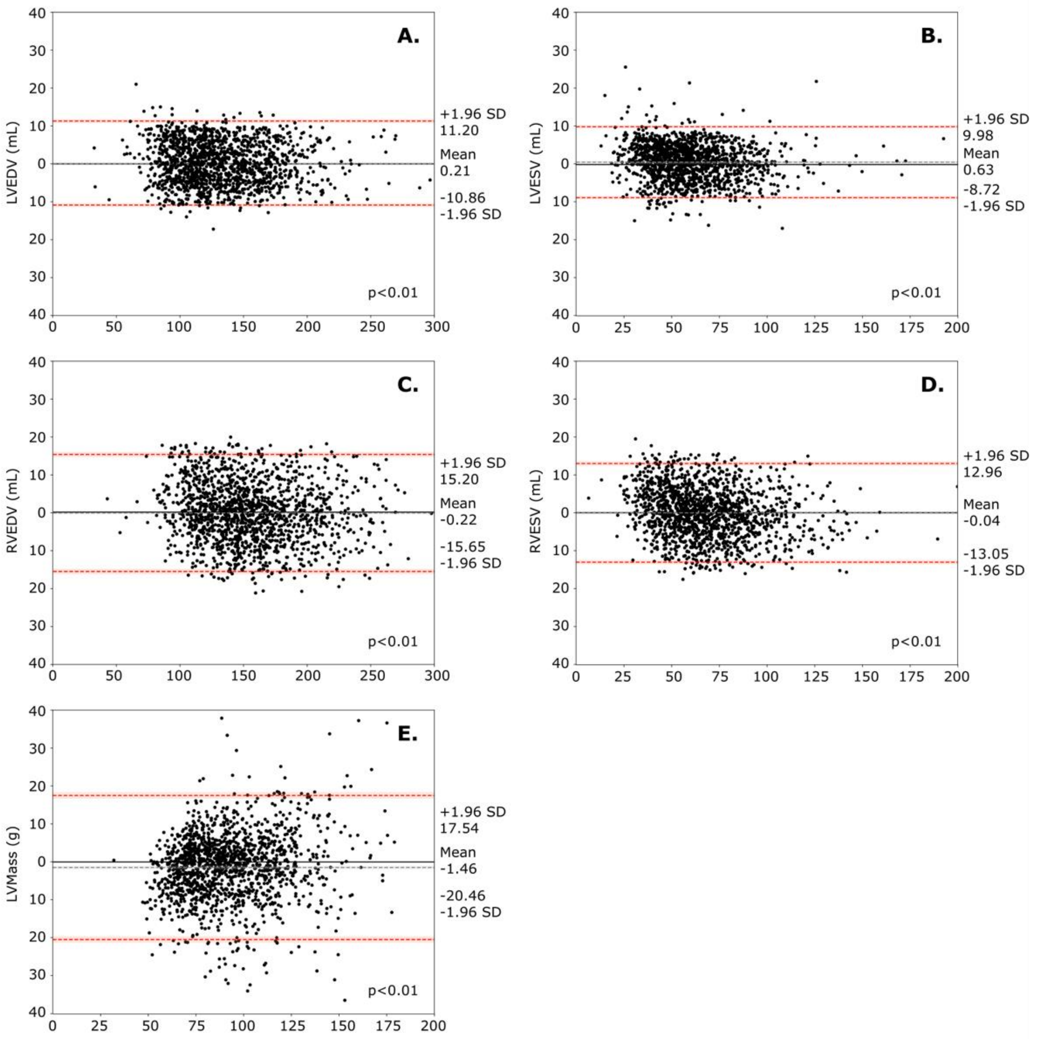

Bland-Altman plots for (A) Left ventricular end-diastolic volume (LVEDV), (B) left ventricular end-systolic volume (LVESV), (C) right ventricular end-diastolic volume (RVEDV), (D) right ventricular end-systolic volume (RVESV), and (E) left ventricular end-diastolic mass (LVM). The grey dotted lines represent the mean bias, and the red dotted lines the limits of agreement. The p values represent the difference in mean bias from zero using a paired *t*-test.

Supplementary Table 1. Associations between average DSC and each racial group

Standardised beta-coefficients (95% CI) and p-value

N=1250	Model 3 - White	p-value	Model 3 - Mixed	p-value	Model 3 - Asian	p-value	Model 3 - Black	p-value	Model 3 - Chinese	p-value	Model 3 - Other	p-value
Age	0.03 (-0.02, 0.08)	0.189	-0.05 (-0.11, 0.00)	0.069	-0.05 (-0.11, 0.00)	0.251	-0.03 (-0.10, 0.03)	0.259	-0.04 (-0.11, 0.02)	0.158	-0.04 (-0.10, 0.02)	0.167
Sex	0.00 (-0.05, 0.06)	0.958	0.09 (0.02, 0.16)*	0.014	0.05 (-0.02, 0.12)	0.203	0.10 (0.04, 0.18)*	0.008	0.07 (-0.01, 0.14)	0.074	0.08 (0.01, 0.15)	0.023
Weight	0.22 (-0.19, 0.66)	0.307	-0.01 (-0.58, 0.57)	0.975	0.13 (-0.45, 0.75)	0.675	0.27 (-0.27, 0.84)	0.340	-0.14 (-0.68, 0.43)	0.630	0.01 (-0.60, 0.62)	0.964
Height	-0.09 (-0.35, 0.17)	0.507	-0.01 (-0.36, 0.36)	0.962	-0.10 (-0.48, 0.29)	0.607	-0.18 (-0.52, 0.17)	0.302	0.09 (-0.26, 0.45)	0.614	-0.04 (-0.42, 0.36)	0.850
BMI	-0.12 (-0.47, 0.20)	0.466	0.06 (-0.40, 0.54)	0.804	-0.07 (-0.57, 0.41)	0.779	-0.19 (-0.65, 0.25)	0.404	0.17 (-0.28, 0.61)	0.466	0.03 (-0.46, 0.53)	0.900
HR	0.03 (-0.01, 0.07)	0.099	0.03 (-0.02, 0.09)	0.234	0.03 (-0.02, 0.09)	0.251	0.01 (-0.04, 0.06)	0.698	0.03 (-0.02, 0.09)	0.193	0.03 (-0.03, 0.08)	0.345
SBP	-0.02 (-0.07, 0.04)	0.511	-0.01 (-0.07, 0.05)	0.733	-0.02 (-0.09, 0.04)	0.506	-0.02 (-0.08, 0.04)	0.625	-0.03 (-0.09, 0.03)	0.434	-0.03 (-0.09, 0.03)	0.315
DBP	-0.03 (-0.08, 0.01)	0.147	-0.01 (-0.07, 0.05)	0.766	-0.01 (-0.08, 0.04)	0.629	-0.01 (-0.07, 0.05)	0.682	-0.03 (-0.08, 0.03)	0.347	-0.01 (-0.06, 0.05)	0.821
LVEDV	-0.03 (-0.25, 0.17)	0.733	-0.15 (-0.43, 0.14)	0.268	-0.16 (-0.45, 0.11)	0.247	-0.15 (-0.42, 0.14)	0.282	-0.13 (-0.41, 0.13)	0.359	-0.14 (-0.41, 0.13)	0.291
LVESV	-0.06 (-0.20, 0.07)	0.367	-0.07 (-0.27, 0.11)	0.442	-0.06 (-0.25, 0.14)	0.521	-0.07 (-0.26, 0.11)	0.418	-0.08 (-0.26, 0.12)	0.440	-0.07 (-0.25, 0.12)	0.454
RVEDV	0.14 (-0.06, 0.36)	0.197	0.25 (-0.07, 0.55)	0.118	0.26 (-0.04, 0.58)	0.085	0.26 (-0.06, 0.58)	0.107	0.22 (-0.10, 0.54)	0.172	0.23 (-0.08, 0.52)	0.135
RVESV	-0.11 (-0.27, 0.03)	0.139	-0.16 (-0.38, 0.05)	0.135	-0.17 (-0.39, 0.04)	0.122	-0.18 (-0.40, 0.03)	0.095	-0.16 (-0.38, 0.06)	0.140	-0.15 (-0.35, 0.07)	0.145
LVmass	-0.02 (-0.09, 0.04)	0.499	-0.01 (-0.08, 0.07)	0.897	0.02 (-0.07, 0.10)	0.641	-0.06 (-0.14, 0.02)	0.184	0.00 (-0.09, 0.08)	0.930	-0.01 (-0.09, 0.08)	0.862
Diabetes	0.08 (-0.10, 0.25)	0.357	0.11 (-0.10, 0.31)	0.264	0.12 (-0.10, 0.34)	0.268	0.19 (-0.01, 0.41)	0.059	0.15 (-0.07, 0.36)	0.172	0.14 (-0.08, 0.36)	0.171
Hypertension	0.06 (0.01, 0.11)	0.030	0.03 (-0.02, 0.09)	0.243	0.04 (-0.02, 0.10)	0.173	0.04 (-0.01, 0.10)	0.109	0.03 (-0.03, 0.09)	0.255	0.05 (-0.01, 0.11)	0.084
Hypercholesterolemia	-0.01 (-0.05, 0.04)	0.799	0.02 (-0.04, 0.08)	0.526	0.00 (-0.06, 0.06)	0.928	0.00 (-0.06, 0.06)	0.963	0.01 (-0.05, 0.07)	0.720	0.01 (-0.05, 0.07)	0.797
Smoking	0.00 (-0.04, 0.04)	1.000	-0.01 (-0.06, 0.04)	0.669	0.00 (-0.05, 0.05)	0.934	-0.01 (-0.05, 0.05)	0.750	0.00 (-0.06, 0.04)	0.874	-0.01 (-0.07, 0.04)	0.584
Centre	0.14 (0.08, 0.20)	0.104	-0.25 (-0.32, -0.18)	0.080	-0.26 (-0.34, -0.18)	0.101	-0.24 (-0.31, -0.17)	0.386	-0.26 (-0.33, -0.20)	0.440	-0.26 (-0.33, -0.19)	0.071
Racial group	-0.74 (-0.80, -0.69)*	0.001	0.23 (0.19, 0.27)**	0.001	0.17 (0.11, 0.21)**	0.001	0.24 (0.20, 0.28)**	0.001	0.21 (0.17, 0.24)**	0.001	0.18 (0.15, 0.21)**	0.001

Standardized regression beta-coefficients and CI, representing the z-score change in variables with increasing DSC. Each model is for a comparison between each racial group vs. all other racial groups (e.g. the third column is the regression analysis for the White group vs. the combination of Mixed, Asian, Black, Chinese and Other groups). LV: left ventricle, EDV: end-diastolic volume, ESV: end-systolic volume, SBP: systolic blood pressure, DBP: diastolic blood pressure; CI: confidence interval. Model 3 is the multivariate linear regression per racial

group adjusted for sex, height, weight, blood pressure at scan-time, heart rate at scan-time, LVEDV, LVESV, RVEDV, RVESV, LVmass, diabetes, hypertension, hypercholesterolemia, smoking and centre. * *$p<.01$, *** *$p<.001$, **** *$p<.0001$*.

Supplementary Table 2. Results of the one-way ANOVA and ANCOVA.

(a) Model 4 (1-way ANOVA)

	F	Sig.	η^2
Racial group	219.43	0.00	0.47

(b) Model 5 (ANCOVA)

	F	Sig.	η^2
Age	1.51	0.13	0.00
Sex	0.61	0.22	0.00
Weight	0.18	0.43	0.00
Height	0.00	0.67	0.00
BMI	0.01	0.98	0.00
HR	1.88	0.93	0.00
SBP	0.31	0.17	0.00
DBP	2.32	0.58	0.00
LVEDV	0.03	0.13	0.00
LVESV	1.05	0.87	0.00
RVEDV	0.95	0.30	0.00
RVESV	1.74	0.33	0.00
LVmass	1.87	0.19	0.00
Diabetes	0.97	0.17	0.00
Hypercholesterolemia	0.03	0.33	0.00
Hypertension	4.85	0.85	0.00
Smoking	0.04	0.03	0.00
Centre	32.18	0.84	0.03
Racial group	167.48	0.00	0.41

Outcomes of the 1-way ANOVA (model 4) and the ANCOVA (model 5) adjusted for sex, height, weight, blood pressure at scan-time, heart rate at scan-time, LVEDV, LVESV, RVEDV, RVESV, LVmass, diabetes, hypertension, hypercholesterolemia, smoking and centre. LV: left ventricle, EDV: end-diastolic volume, ESV: end-systolic volume, SBP: systolic blood pressure, DBP: diastolic blood pressure.

Supplementary Table 3. Derived p-values of the Student's *t*-test for the Dice similarity coefficient (DSC) values for the overall test set and by sex and race

N=1,250	LVBP		LVMyo		RVBP		AVG	
**Male/ Female**	0.613		0.104		0.196		0.998	
**White/Non-White**	2.47E-06	***	2.27E-06	***	8.57E-07	***	8.06E-07	***
**Mixed/Non- Mixed**	5.85E-06	**	7.35E-06	**	9.04E-06	**	5.68E-06	**
**Asian/Non- Asian**	5.53E-06	**	3.86E-05	*	2.68E-05	**	3.11E-05	**
**Black/Non- Black**	1.45E-06	***	4.79E-07	***	1.59E-06	***	4.91E-07	***
**Chinese/Non- Chinese**	1.48E-04	*	9.28E-05	*	1.49E-04	*	1.65E-04	*
**Others/Non- Others**	2.13E-04	*	3.53E-04	*	3.96E-04		1.91E-04	*

Computed p-values of Table 2, which compares DSC between groups (i.e. male vs. female, white vs. non-white, mixed vs. non-mixed, etc) using a Student's *t*-test with Bonferroni correction. The first column of each group shows the exact p-value and the second column shows statistical significance after Bonferroni correction (28 tests): * $p<.01/28$, ** $p<.001/28$, *** $p<.0001/28$. LVBP: LV blood pool, LVMyo: LV myocardium, RVBP: RV blood pool, and AVG: average across LVBP, LVM and RVBP.

Supplementary Table 4. Derived p-values of the Student's *t*-tests for the manual clinical measures (top table) and absolute (middle table) and relative (bottom table) differences in volumetric and functional measures between automated and manual segmentations, overall and by sex and race.

(a) Manual

	iLVEDV (mL/mm2)	iLVESV (mL/mm2)	LVEF (%)	iLVmass (g/mm2)	iRVEDV (mL/mm2)	iRVESV (mL/mm2)	RVEF (%)
Male/Female	7.91E-06 *	6.01E-01	1.37E-05 *	3.26E-03	4.30E-05 *	6.67E-01	3.66E-08 *
White/Non-White	5.33E-02	4.95E-02	8.81E-01	5.60E-05 *	9.25E-05 *	1.09E-04 *	2.31E-04
Mixed/Non-Mixed	3.60E-05 *	2.62E-05 *	9.60E-05 *	6.56E-01	1.87E-04 *	2.40E-05 *	5.53E-05 *
Asian/Non-Asian	1.07E-04 *	2.99E-05 *	4.64E-05 *	5.08E-05 *	4.97E-05 *	1.25E-02	4.38E-02
Black/Non-Black	2.71E-03	2.48E-01	1.69E-01	9.78E-01	1.19E-04 *	6.99E-02	2.76E-01
Chinese/Non-Chinese	1.77E-04 *	2.24E-05 *	4.17E-05 *	1.90E-04 *	2.16E-02	7.50E-02	3.45E-02
Others/Non-Others	2.06E-05 *	7.14E-03	1.68E-04 *	7.06E-01	2.68E-01	4.50E-02	2.34E-02

(b) Absolute difference

	iLVEDV (mL/mm2)	iLVESV (mL/mm2)	LVEF (%)	iLVmass (g/mm2)	iRVEDV (mL/mm2)	iRVESV (mL/mm2)	RVEF (%)
Male/Female	2.43E-01	5.71E-02	6.99E-05 *	1.10E-02	2.97E-01	1.08E-01	5.71E-05 *
White/Non-White	5.20E-01	3.53E-05 *	2.84E-06 *	1.36E-04 *	5.03E-06 *	2.21E-02	2.66E-06 *
Mixed/Non-Mixed	1.54E-04 *	1.80E-05 *	2.36E-04	1.54E-04 *	6.64E-05 *	7.35E-05 *	3.89E-05 *
Asian/Non-Asian	2.29E-05 *	1.52E-04 *	2.15E-04	2.98E-05 *	2.60E-05 *	4.10E-03	1.16E-01

Black/Non-Black	3.42E-05	*	5.26E-05	*	1.96E-04	*	3.64E-05	*	2.86E-05	*	5.89E-01		2.02E-01	
Chinese/Non-Chinese	2.50E-05	*	1.14E-05	*	9.16E-05	*	1.93E-04	*	6.72E-03		3.59E-05	*	3.88E-05	*
Others/Non-Others	2.29E-04		2.81E-05	*	4.91E-05	*	8.28E-03		2.08E-01		1.67E-04	*	5.36E-02	

(c) Relative difference

	iLVEDV (mL/mm2)		iLVESV (mL/mm2)		LVEF (%)		iLVmass (g/mm2)		iRVEDV (mL/mm2)		iRVESV (mL/mm2)		RVEF (%)	
Male/Female	2.43E-06	*	2.97E-06	*	1.39E-04	*	5.71E-05	*	1.08E-06	*	6.99E-05	*	5.71E-05	*
White/Non-White	5.20E-05	*	5.03E-05	*	2.30E-01		3.53E-01		2.21E-05	*	2.84E-01		2.66E-06	*
Mixed/Non-Mixed	1.54E-04	*	6.64E-05	*	5.25E-06	*	1.80E-05	*	7.35E-05	*	2.36E-05	*	3.89E-05	*
Asian/Non-Asian	2.29E-06	*	2.60E-06	*	3.65E-08	*	1.52E-04	*	4.10E-05	*	2.15E-04		1.16E-01	
Black/Non-Black	3.42E-02		2.86E-02		2.28E-09	*	5.26E-01		5.89E-01		1.96E-01		2.02E-01	
Chinese/Non-Chinese	2.50E-05	*	6.72E-05	*	9.08E-05	*	1.14E-04	*	3.59E-02		9.16E-05	*	3.88E-05	*
Others/Non-Others	2.29E-05	*	2.08E-04		3.12E-05	*	2.81E-03		1.67E-02		4.91E-05	*	5.36E-02	

Computed p-values of Table 3, which compares cardiac volumes between groups (i.e. male vs female, white vs non-white, mixed vs non-mixed, etc) using a Student's *t*-test with Bonferroni correction. The first column of each group shows the exact p-value and the second column shows an asterisk for the cases that are statistically significant after correction (49 tests), i.e. $p<0.01/49$. LV: left ventricle, RV: right ventricle, EDV: end diastolic volume, ESV: end systolic volume, EF: ejection fraction, LVMass: left ventricular mass.
